# Updates in Pathology for Retroperitoneal Soft Tissue Sarcoma

**DOI:** 10.3390/curroncol29090504

**Published:** 2022-09-07

**Authors:** Tanner Mack, Bibianna Purgina

**Affiliations:** 1 Department of Pathology and Laboratory Medicine, The Ottawa Hospital, University of Ottawa, Ottawa, ON K1H 8L6, Canada; 2 Eastern Ontario Regional Laboratory Association (EORLA), Ottawa, ON K1H 8L6, Canada

**Keywords:** retroperitoneum, sarcoma, molecular, liposarcoma, leiomyosarcoma, GIST, malignant peripheral nerve sheath tumor, solitary fibrous tumor, pathology

## Abstract

Retroperitoneal tumors are extremely rare. More than 70% of primary retroperitoneal soft tissue tumors are malignant. The most common sarcomas in the retroperitoneum include liposarcomas and leiomyosarcoma, however other sarcomas, along with benign mesenchymal tumors, can occur. Sarcomas are a heterogenous group of tumors with overlapping microscopic features, posing a diagnostic challenge for the pathologist. Correct tumor classification has become important for prognostication and the evolving targeted therapies for sarcoma subtypes. In this review, the pathology of retroperitoneal soft tissue sarcomas is discussed, which is important to the surgical oncologist. In addition, less common sarcomas and benign mesenchymal tumors of the retroperitoneum, which may mimic sarcoma clinically and pathologically, are also discussed.

## 1. Introduction

Sarcomas are a rare type of malignancy derived from primitive multipotential mesenchymal precursors. They represent less than 1% of all malignant tumors and are broadly categorized into bone or soft tissue [1]. Soft tissue tumors (STTs) can be benign, intermediate (locally aggressive or rarely metastasizing), or malignant. Soft tissue sarcomas (STSs) include both intermediate and malignant categories and are more commonly seen in the adult population. The etiology of most STTs remains unknown. Less than 10% of cases can be attributed to genetics, environmental factors, irradiation, viral infections or immunodeficiency. The majority of cases seem to arise de novo without an apparent causative factor. There is a slight male predominance and approximately 10% of patients have detectable metastatic disease (frequently to the lungs) at the time of primary diagnosis. The most common anatomic sites for STS are the extremities (75%) followed by the trunk wall (10%), and retroperitoneum (10%), with all other sites representing <5% of cases [1].

It is important to recognize that many tumors other than sarcomas can occur in the retroperitoneum. Specifically, retroperitoneal organs, such as the large bowel, kidneys, adrenal glands, pancreas, ureters, large vessels, nerves, and lymph nodes, can all develop primary diseases specific to that organ. Additionally, retroperitoneal lymph node metastases can present as a retroperitoneal mass. Malignant tumors of the retroperitoneum are roughly four times more frequent than benign lesions [2,3]. The histologic subtype of tumors found within the retroperitoneum/peritoneum are less frequently sarcomas (40.8%) compared to all other histotypes (59.2%), which include carcinomas, melanomas, or lymphomas [4].

Retroperitoneal tumors are often much larger before they become symptomatic and, therefore, tend to present at a later stage, resulting in lower survival rates compared to tumors of the extremities. The current standard of treatment for retroperitoneal sarcomas involves complete surgical excision with negative margins. The most important prognostic factors for survival are completeness of surgical resection, histological type/subtype, and grade [1,5,6,7]. The FNCLCC (Fédération Nationale des Centres de Lutte Contre le Cancer) system is the preferred method for sarcoma tumor grading. This three-tiered grading system stratifies tumors by the degree of necrosis, mitotic activity, and differentiation.

The 2020 World health organization (WHO) 5th edition of Soft tissue and bone tumors recognizes more than 100 histologic types of STT that are grouped into 12 categories, based on the following tumor cell lineages: (1) adipocytic, (2) fibroblastic/myofibroblastic, (3) fibrohistiocytic, (4) vascular, (5) pericytic, (6) smooth muscle, (7) skeletal muscle, (8) gastrointestinal stromal tumors, (9) chondro-osseus, (10) peripheral nerve sheath, (11) tumors of uncertain differentiation, and (12) undifferentiated small round cell sarcomas [1]. Despite this morphologic classification, the cells of origin for most sarcomas are not well understood and are presumed to arise from mesenchymal derived stem cell precursors. In recent years, extensive molecular profiling of sarcomas has identified characteristic genetic alterations, including unique translocations, oncogene activations, loss of tumor suppressors, and copy number variations [8]. Overall, STSs are a diverse group of tumors with significant morphologic overlap.

We review in detail the three most common primary retroperitoneal sarcomas: (1) well-differentiated liposarcoma (WDLPS), (2) dedifferentiated liposarcoma (DDLPS), and (3) leiomyosarcoma (LMS). This review is then followed by a discussion of other less common tumors that can arise in the retroperitoneum, including pleomorphic liposarcoma (PLPS), undifferentiated pleomorphic sarcoma (UPS), malignant peripheral nerve sheath tumor (MPNST), desmoplastic small round cell tumor (DSRCT), Ewing sarcoma, solitary fibrous tumor (STF), gastrointestinal stromal tumor (GIST), inflammatory myofibroblastic tumor (IMT), angiomyolipoma (AML), myelolipoma, and chordoma.

## 2. Liposarcoma (LPS)

LPSs are a heterogenous group of malignant neoplasms derived from an adipocytic cell lineage. The WHO outlines five types of LPS, including WDLPS/atypical lipomatous tumor (ALT), DDLPS, myxoid LPS, PLPS, and myxoid pleomorphic LPS [1]. The vast majority of retroperitoneal LPSs are either WDLPS or DDLPS [7,9,10,11,12]. Four independent prognostic factors affecting overall survival in retroperitoneal LPSs include age, tumor site, tumor necrosis, and recurrence [11]. There is no definitive evidence to support the routine use of adjuvant radiation therapy (RT) or adjuvant chemotherapy [13]. Anthracycline-based chemotherapy regimens, such as doxorubicin, may be indicated for advanced or metastatic LPS [7]. The role of neoadjuvant RT has recently been prospectively evaluated in a randomized controlled trial (EORTC-62092: STRASS) which concluded that the routine use of neoadjuvant RT is not recommended in patients with retroperitoneal sarcomas of any grade; however post-hoc sub-group analysis suggested that patients with LPS may benefit from neoadjuvant RT [14]. Additionally, a prospective randomized study (EORTC-1809: STRASS II) is currently investigating the role of neoadjuvant chemotherapy in high-grade retroperitoneal sarcomas, including DDLPS and LMS.

### 2.1. Well-Differentiated Liposarcoma (WDLPS)

WDLPS and ALT are synonymous terms describing morphologically and genetically similar malignant neoplasms. By convention, ALT is used to describe lesions arising at anatomical sites where surgical resection is curative, such as the extremities. WDLPS is reserved for tumors which reside within body cavities, such as the retroperitoneum. WDLPS/ALT is a locally aggressive mesenchymal neoplasm without metastatic potential. It accounts for 40–45% of all liposarcomas [1]. Males and females are affected equally with a peak incidence occurring between the fourth and fifth decades of life. The most frequent sites of involvement are the extremities, followed by the retroperitoneum and para-testicular soft tissue. Clinically, retroperitoneal WDLPS usually remains asymptomatic until the tumor has exceeded 20 cm in size. Rarely, retroperitoneal LPS can exceed 30 cm [15]. Most tumors are sporadic in nature, although a few cases can be associated with germline mutations in the TP53 gene, as seen in Li-Fraumeni syndrome.

**Figure 1:**
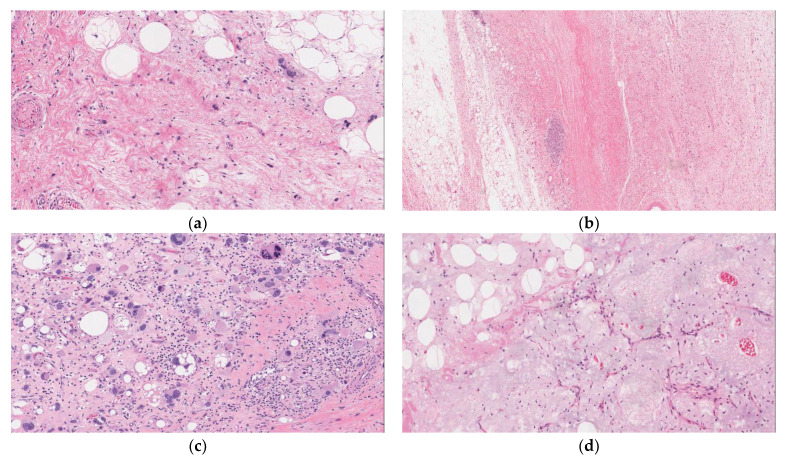
Liposarcoma subtypes. Photomicrographs from representative cases obtained at The Ottawa Hospital. Slides stained with Hematoxylin and Eosin (H&E) with corresponding magnifications as follows: Well-differentiated liposarcoma (WDLPS) with hyperchromatic and atypical nuclei within adipocytes and fibrous septa H&E 100× (**a**) De-differentiated liposarcoma (DDLPS) with transition from lipomatous component to non-lipomatous solid component H&E 20× (**b**) pleomorphic liposarcoma with pleomorphic multi-vacuolated lipoblasts H&E 100× (**c**) myxoid liposarcoma H&E 100× (**d**).

WDLPSs are well circumscribed yellow lobulated lesions that are rarely infiltrative. When localized to the retroperitoneum they may present as multiple discontinuous masses. WDLPSs/ALTs are categorized into three subtypes listed in decreasing order of frequency: adipocytic (lipoma-like), sclerosing, and inflammatory [16]. Mixed subtypes also occur, particularly in the retroperitoneum [17,18]. Histologically, tumors are composed of variably sized adipocytes with intervening thick fibrous septae and scattered thick-walled blood vessels. Adipocytes and stromal cells exhibit focal nuclear atypia and hyperchromasia, which are normally absent in benign lipomas (Figure 1a). Lipoblasts may also be appreciated; however, their presence is not required for the diagnosis [1]. Rarely, heterologous elements, such as osseous (bone), smooth muscle, or striated muscle, may also be identified but this in and of itself should not imply de-differentiation [16,19,20,21]. Increased fibrosis or inflammation may also be appreciated in variable quantities and warrants classification of the aforementioned subtype if the specific component represents the majority of the tumor.

Molecular alterations of WDLPS are characterized by a supernumerary ring and giant marker chromosomes with amplified sequences from the 12q14-15 region (notably containing *MDM2* and *CDK4* genes among others) [22,23,24]. Ancillary studies that are frequently used to help support the diagnosis of WDLPS include immunohistochemistry to stain for the nuclear expression of MDM2 or CDK4 proteins, or fluorescence in-situ hybridization (FISH) to quantify the corresponding gene amplifications [25,26,27]. Such ancillary studies are common adjuncts in the work up of LPS, particularly in difficult cases. However, they are not essential for the diagnosis of WDLPS, which can frequently be made by morphology alone.

The most important prognostic factor for WDLPS/ALT is anatomic location. Tumors located in deep sites, such as the retroperitoneum, tend to recur repeatedly [1]. The median time to death for all WDLPS/ALT ranges from 6 to 11 years. When considering retroperitoneal WDLPS, the overall risk of de-differentiation is >20%, and the overall mortality is >80% for patients that are followed for 10–20 years [1]. 

Benign entities that should be considered in the differential diagnosis of WDLPS are lipomas and their variants, hibernoma, and idiopathic retroperitoneal fibrosis. Lipomas are extremely rare in the retroperitoneum and caution should be made when making this diagnosis [28]. The diagnostic features of focal nuclear atypia and hyperchromasia can easily be missed due to small biopsies and sampling bias. Recommendations for ordering FISH have been established to help decrease the risk of underdiagnosing WDLPS in lipomatous lesions. Specifically, recurrent lesions, deep (below fascia) extremity lesions that are more than10 cm in patients older than 50 years of age, cases with equivocal cytologic atypia, and lesions of the retroperitoneum/pelvis/abdomen should all undergo FISH analysis for *MDM2* amplification [29]. 

Other malignant entities to consider in the differential include fat forming SFT, and other subtypes of LPS, such as DDLPS or myxoid LPS. WDLPS and DDLPS may occasionally show extensive areas with myxoid change which can mimic a myxoid LPS (Figure 1d). Molecular testing can be used to differentiate these entities; specifically, myxoid LPS are characterized by a *FUS-DDIT3* translocation [1]. Additionally, myxoid LPSs more commonly arise in the extremities and are very rarely diagnosed in the retroperitoneum. Most reported cases of retroperitoneal myxoid LPS represent metastatic disease from other soft tissue sites [30]. To ensure correct classification, adequate sampling of the tumor at the time of grossing is essential, as any dedifferentiated components that are identified portend a worse prognosis [11,12,31]. This issue may also be encountered due to sampling bias on small biopsies that show no areas of dedifferentiation leading to an incorrect classification until the excised specimen has been fully examined [32]. Finally, WDLPS with an inflammatory subtype can bring into the differential non-adipocytic lesions, such as inflammatory myofibroblastic tumor, Castleman disease, and lymphomas.

### 2.2. De-Differentiated Liposarcoma (DDLPS)

DDLPS is a higher grade, often non-lipogenic, sarcoma with metastatic potential that is genetically similar to WDLPS/ALT. It can occur de novo within, or adjacent to, a preexisting WDLPS that has undergone dedifferentiation, or it can be seen without any identifiable well-differentiated components. Interestingly, local recurrence from a previous DDLPS may consist entirely of the well-differentiated component. DDLPS may also recur from a previously resected pure WDLPS. De-differentiation occurs in up to 10% of all WDLPS/ALT; however, as previously mentioned, the risk is increased to >20% for tumors located in the retroperitoneum [1]. This likely represents a time-dependent, as opposed to site-dependent, phenomenon. Approximately 90% of DDLPSs arise de novo and 10% develop as recurrences. Overall, the retroperitoneum is the most common site of DDLPS and it is approximately 10 times more frequent at this location than any other somatic soft tissue site [1].

Surgically, the non-lipomatous (dedifferentiated) components are generally easy to identify. However, the well-differentiated components may be more challenging to distinguish from normal fat; therefore, complicating complete surgical removal. The transition between well-differentiated and de-dedifferentiated components may be abrupt or gradual, therefore extensive sampling of the tumor is essential. 

Histologically, the hallmark of DDLPS is the transition from WDLPS into a typically non-lipomatous sarcoma composed of fibroblastic spindle cells arranged in a fascicular pattern with higher grade nuclear atypia (Figure 1b). Dedifferentiated areas frequently resemble UPS or myxofibrosarcoma. Additionally, DDLPS may show heterologous differentiation (frequently myogenic) in approximately 5–10% of cases [33]. Specifically, rhabdomyoblastic differentiation appears to be associated with worse outcomes [34]. Immunohistochemistry is mainly used to confirm divergent differentiation and to exclude other tumor types.

The molecular features of DDLPS overlap with WDLPS [22,23,24]. They are both defined by *MDM2* and *CDK4* amplifications that can be confirmed by immunohistochemistry and FISH for *MDM2* overexpression, thereby helping to distinguish it from PLPS and myxoid LPS. With that said, the overall genomic landscape of DDLPS is often more complex than WDLPS. One genetic difference is the co-amplification of 1p32 and 6q23 which are present in DDLPS and never seen in WDLPS [23]. The genes in these regions are, therefore, likely to be involved in the dedifferentiation process.

In both WDLPS and DDLPS the overexpression of *MDM2* targets p53 for degradation, thereby interfering with tumor suppressor pathways within the cell cycle. Targeting the MDM2-p53 axis is an enticing chemotherapeutic strategy, and several drugs have been developed to block this interaction; however, many early phase clinical studies have reported only partial patient responses and multiple adverse events limiting their clinical use [22,35]. A current clinical trial is also investigating the combined effect of MDM2 inhibition in combination with RT [36].

The most important prognosticator for DDLPS is anatomic site, with retroperitoneal sites having an overall worse prognosis. Local recurrence is seen in up to 40% of cases. However, essentially all retroperitoneal LPSs tend to recur if patients are followed for 10–20 years [1]. Identification of a dedifferentiated component at the resections margins in retroperitoneal DDLPS is associated with a shorter local recurrence free survival [37]. Distant metastases are observed in 15–20% of cases and overall mortality is ~30% at 5 years, although the rates are much higher when patients are followed for 10–20 years [1]. The extent of tumor dedifferentiation does not appear to predict a worse outcome, and, despite its high-grade morphology, DDLPS exhibits a less aggressive clinical course than other high-grade sarcomas. Surgically, multi-visceral resections appear to improve relapse free survival for both WDLPS and DDLPS [38]. The combined features of a WDLPS and non-lipogenic sarcomatous component narrows the differential diagnosis considerably. However, without a convincingly identifiable well-differentiated component other diagnostic entities that should be considered include an UPS, myxofibrosarcoma, PLPS, pleomorphic LMS, and MPNST.

### 2.3. Pleomorphic Liposarcoma (PLPS)

Pleomorphic liposarcoma (PLPS) is a high-grade sarcoma with no identifiable areas of WDLPS/ALT (confirmed by the absence of *MDM2* gene alterations) or other lines of differentiation. PLPS has a male predominance and a peak incidence in the seventh decade of life. It represents less than 5% of all LPSs with most cases occurring in the extremities, followed by the trunk wall, retroperitoneum, and spermatic cord [1].

PLPSs are usually well demarcated non-encapsulated masses. Microscopically, the tumors are composed of high-grade cells with varying numbers of pleomorphic and bizarre multinucleated tumor cells (Figure 1c). Pleomorphic lipoblasts are required for the diagnosis; however, their presence is variable and can be missed in small biopsies or inadequately sampled tumors. Necrosis is present in more than half of cases. An epithelioid morphology can be seen in approximately 25% of cases [1]. Immunohistochemistry frequently shows nonspecific staining for most markers; however, the epithelioid subtype can show focal positive staining for cytokeratin’s or Melan-A. Molecular studies of PLPS differ from other LPSs by having no consistent cytogenetic abnormalities [23,24]. PLPS is an aggressive tumor with an overall 5-year survival of approximately 60%. Local recurrence and metastasis are seen in 30–50% of cases [1]. Poor prognostic factors include tumors located within the retroperitoneum, increased tumor depth, size, and mitotic rate [11,12]. The differential diagnosis includes myxofibrosarcoma, UPS, pleomorphic rhabdomyosarcoma, DDLPS, and myxoid LPS.

## 3. Leiomyosarcoma (LMS)

LMSs are tumors with smooth muscle differentiation. They are the most frequent sarcoma subtype and account for approximately 11% of all newly diagnosed STSs [1]. Although most cases arise in the uterus, other common sites of involvement include the extremities, retroperitoneum, abdomen/pelvis, large vessels, and trunk. Vascular LMS typically arises from the inferior vena cava and poses a unique treatment challenge, often requiring the expertise of a vascular surgeon. The risk of LMS increases with age and peaks around the seventh decade of life. There is an equal prevalence between men and women, except for cases that arise in the retroperitoneum or vena cava, which more frequently affect women [1].

**Figure 2:**
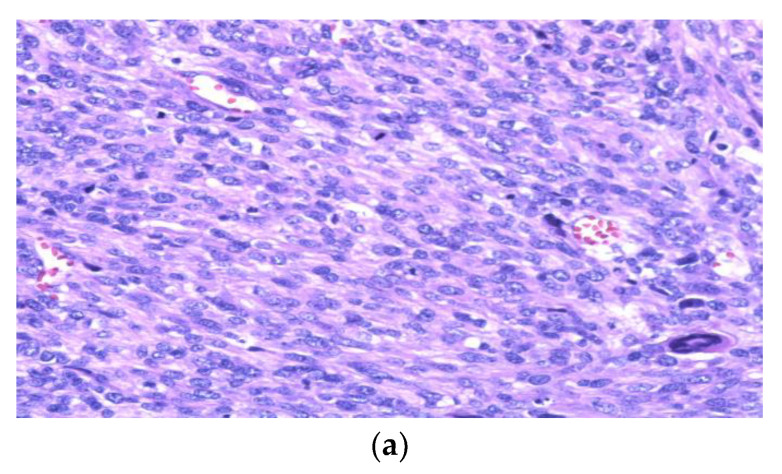

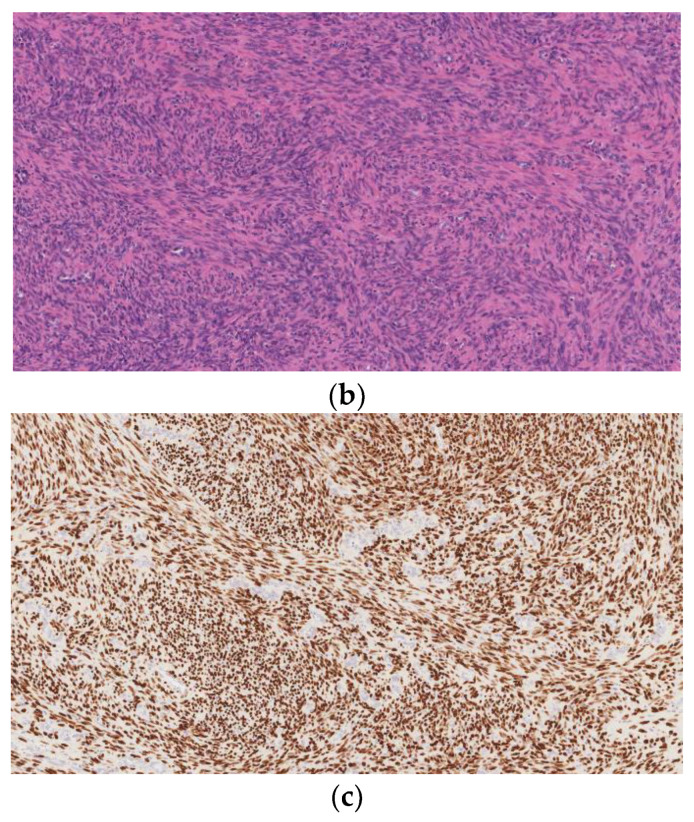
Photomicrographs from representative cases obtained at The Ottawa Hospital. Slides stained with Hematoxylin and Eosin (H&E) or in situ hybridization (ISH) with corresponding magnifications as follows: Leiomyosarcoma H&E 100× (**a**) Ebstein-Barr virus-associated smooth muscle tumor (EBV-SMT) H&E 100× (**b**) EBV-SMT positive for EBER (EBV-encoded small RNA) ISH 100× (**c**).

LMSs are usually firm white rubbery masses with a whorled appearance similar to benign leiomyomas. When LMSs are poorly differentiated they tend to have a softer and fleshy appearance with variable regions of necrosis, hemorrhage, or cystic change. Such changes are also more frequently seen in larger tumors. Microscopically, LMS is composed of long intersecting fascicles of plump spindle cells (Figure 2a). The cytologic features show blunt ended, cigar shaped, nuclei with moderate amounts of eosinophilic cytoplasm. Moderate nuclear pleomorphism is usually noted, along with easily identifiable mitotic figures with atypical forms. The majority of LMSs are high-grade and show a well circumscribed border. However, infiltrative tumors can also be seen. Lymph node metastases are very rare. Immunohistochemistry normally stains the malignant cells with at least one myogenic marker (i.e., smooth muscle actin, desmin, or h-caldesmon), and >70% of cases show positivity for more than one of these markers [1]. Extra-uterine LMSs are frequently estrogen and progesterone receptor negative, compared to the positive expression commonly seen in uterine LMS [39].

Ebstein-Barr virus-associated smooth muscle tumors (EBV-SMT) are rare and under recognized smooth muscle neoplasms of uncertain malignant potential that have been described throughout the body, including the retroperitoneum and abdominal cavity [40,41,42,43]. They develop in immunosuppressed patients from a variety of causes, including HIV infection and post-transplant immunosuppression. Morphologically, they resemble smooth muscle neoplasms (Figure 2b). A clinical history of immunosuppression and demonstration of EBV with in situ hybridization for EBER (EBV-encoded small RNA) helps to distinguish an EBV-associated smooth muscle tumor from a LMS (Figure 2c).

The most important prognostic factors for LMS are histological grade, tumor location, and size. Unfortunately, core needle biopsies have been shown to correlate poorly with the final tumor grade, which may complicate therapeutic management [32,44]. Retroperitoneal LMSs are often fatal. Typically, they are large (>10 cm), and difficult, or impossible, to excise with negative (clear) surgical margins. Therefore, retroperitoneal LMSs are prone to both local recurrence and metastasis. Interestingly, LMS is the most common sarcoma to give rise to metastasis to the skin [45]. Distinction between low grade LMS and benign smooth muscle neoplasms should be assessed by the degree of atypia, mitosis and necrosis [46]. Other malignant entities that can show muscle differentiation should also be included in the differential, such as DDLPS, GIST, AML, and UPS.

## 4. Undifferentiated Pleomorphic Sarcoma (UPS)

**Figure 3:**
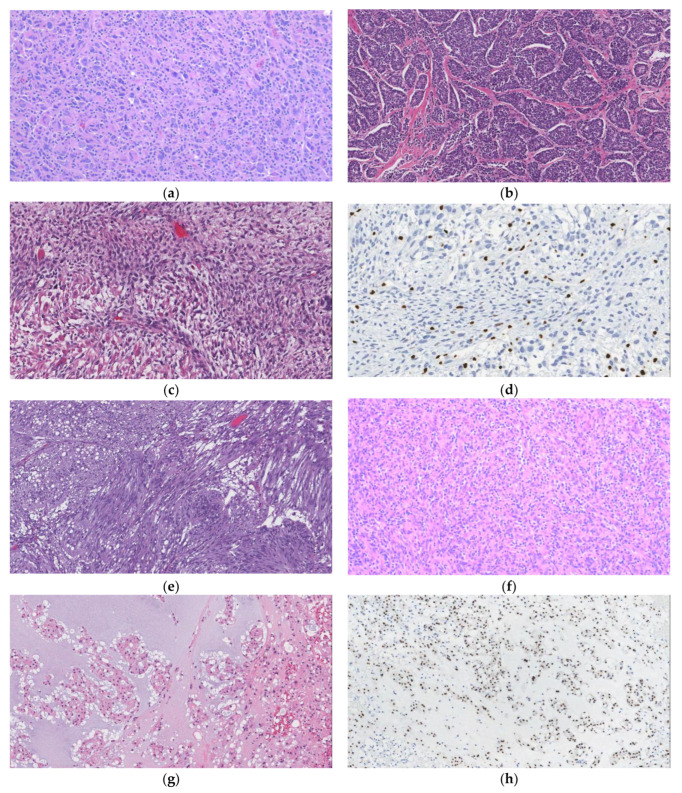

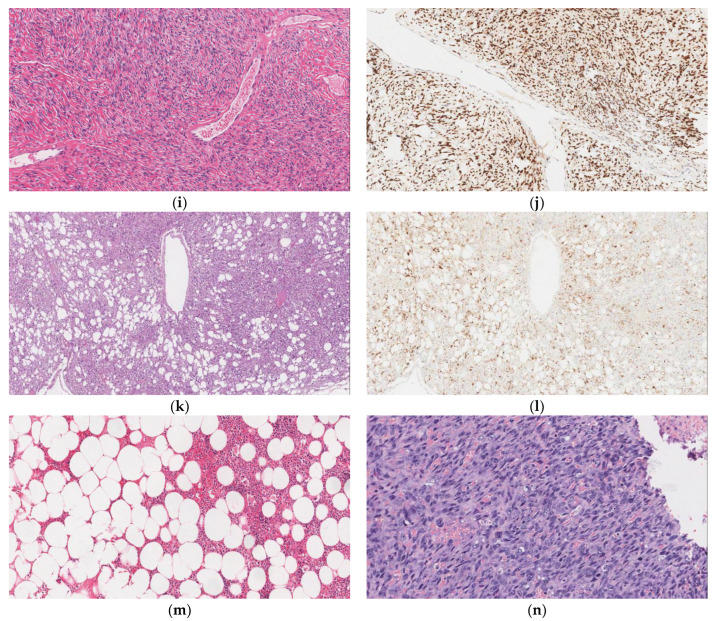
Photomicrographs from representative cases obtained at The Ottawa Hospital. Slides stained with Hematoxylin and Eosin (H&E) or immunohistochemistry (IHC) peroxidase as follows: Undifferentiated pleomorphic sarcoma (UPS) H&E 100× (**a**), Ewing sarcoma H&E 100× (**b**) malignant peripheral nerve sheath tumor (MPNST) with rhabdomyoblastic differentiation “Trition tumor” H&E 200× (**c**) MPNST with H3K27me3 loss IHC 200× (**d**) gastrointestinal stromal tumor (GIST) H&E 100× (**e**) inflammatory myofibroblastic tumor (IMT) H&E 100× (**f**) conventional chordoma H&E 100× (**g**) conventional chordoma with brachyury expression IHC 100× (**h**) solitary fibrous tumor (SFT) H&E 100× (**i**) SFT with STAT6 expression IHC 100× (**j**) Angiomyolipoma (AML) H&E 40× (**k**) AML with HMB45 expression IHC 40× (**l**) myelolipoma H&E 100× (**m**) angiosarcoma H&E 200× (**n**).

Undifferentiated soft tissue sarcomas (USTSs) are high-grade tumors with no distinctive morphologic, immunohistochemical, or molecular features to suggest a specific line of differentiation. They account for up to 20% of all STSs and tend to occur at all ages with no difference between sexes [1]. It is a diagnosis of exclusion, and the etiology remains unknown. However, up to 25% of cases are associated with prior radiation exposure. Specific subtypes include spindle cell, pleomorphic, round cell, and epithelioid. Undifferentiated pleomorphic sarcoma (UPS) represents the most frequent type and was previously classified as malignant fibrous histiocytoma. Histologically, there are no distinctive macroscopic features, other than a pattern-less architecture, extensive pleomorphism with bizarre multinucleated tumor giant cells, abundant mitotic figures with atypical forms, and frequent necrosis and hemorrhage (Figure 3a). Immunohistochemistry shows nonspecific, to negative, staining for most markers in the majority of cases.

Molecular studies have shown no diagnostic finding aside from extensive genomic rearrangements and complex karyotypes [47]. UPSs in adults typically arise in the limbs or trunk and have a reported 5-year metastasis-free survival rate of 83% [48]. USTSs with an epithelioid appearance tend to be more aggressive. Advanced UPSs have the worst outcome compared with other histologic STS subtypes [49]. The differential diagnosis includes poorly differentiated carcinomas, melanoma, DDLPS, pleomorphic LMS, pleomorphic rhabdomyosarcoma, and pleomorphic MPNST.

## 5. Malignant Peripheral Nerve Sheath Tumor (MPNST)

MPNST is a malignant spindle cell neoplasm with neuroectodermal differentiation. It accounts for approximately 3–5% of all STSs [1]. MPNSTs commonly arise in three distinct settings: (1) from a peripheral nerve, typically a major nerve trunk, (2) from malignant transformation of a pre-existing benign nerve sheath tumor, such as a neurofibroma, or (3) in a patient with neurofibromatosis type 1 (NF1). The most common MPNST sites are the trunk and extremities, followed by the head and neck. Specifically, 70% arise from major nerve trucks, most often the sciatic nerve as well as the brachial or sacral plexus, or paraspinal nerves, the latter of which can give rise to retroperitoneal tumors; however, this is a relatively infrequent location. Sporadic and NF1-associated tumors occur in roughly equal proportions [1]. Sporadic cases are typically seen in patients aged 20–50 years old; however, patients with NF1 are usually diagnosed a decade earlier. For patients with NF1, the lifetime risk of developing MPNST is around 2–10%. Another risk factor for MPNST is prior radiation which is attributed to ~10% of sporadic cases [1].

Conventional MPNSTs are large fusiform masses that arise from an adjacent or preexisting nerve. Tumors may also originate from multiple interconnecting nerves and are classified as plexiform, having the gross appearance of a “bag of worms”. Plexiform neurofibroma is considered pathognomonic in patients with NF1. MPNSTs are usually >5 cm at the time of diagnosis and have a firm white-grey cut surface with variable areas of hemorrhage and necrosis. Microscopically, they are composed of relatively uniform spindle cells arranged in fascicles with a herringbone architecture. They can have alternating cellular to hypocellular regions with perivascular accentuation resulting in a marbled appearance. There are often areas of geographic necrosis and conspicuous mitotic figures. Most tumors are histologically high grade. Occasionally cells may show focal epithelial or round cell morphology or extensive pleomorphism. Epithelioid MPNST is a rare subtype representing <5% of tumors and is not associated with NF1 [1]. Heterologous elements (i.e., angiosarcomatous or rhabdomyosarcomatous) can be seen in about 15% of cases; specifically, when skeletal muscle (rhabdomyosarcomatous) differentiation is identified, the neoplasm is referred to as a Triton tumor (Figure 3c) [1]. The immunohistochemical profile for conventional MPNST is usually patchy and focally positive for SOX10 and S-100 protein. Diffuse staining with these markers is not usually compatible with the diagnosis of conventional MPNST. This staining pattern is different from melanoma, which is normally diffusely positive for SOX10 and S100, along with more specific melanoma markers, including Melan-A and HMB45. Additionally, the loss of staining with the antibody H3K27me3 is a new sensitive and specific marker for conventional high grade MPNST (Figure 3d). However, retained staining can also be seen in lower grade tumors [50,51,52]. H3K27me3 is an epigenetic DNA packaging protein that modifies histone H3 by tri-methylating the 27th lysine residue. The staining pattern of epithelioid MPNSTs diverges from the conventional immunophenotype; specifically, epithelioid MPNST shows diffuse staining with SOX10 and S-100, with retained H3K27me3. In addition, most cases show *SMARCB1* gene inactivation, resulting in loss of staining with the corresponding antibody labeled INI1, which is observed in approximately 75% of epithelioid MPNST [53].

MPNST is an aggressive tumor with a poor prognosis. Adverse prognostic factors include truncal location, tumor size >5 cm, local recurrence, and high-grade morphology. Patients with NF1-associated MPNST appear to have a worse prognosis than sporadic tumors. Malignant Triton tumors are particularly aggressive. Overall, 5-year survival is around 50% in sporadic cases to 10–15% in NF1 patients [54]. The differential diagnosis includes entities such as cellular schwannoma, monophasic synovial sarcoma, spindle cell melanoma, DDLPS, LMS, and rhabdomyosarcoma.

## 6. Small Round Blue Cell Tumors

Small round blue cell tumors are a morphologic classification of malignant neoplasms that have a broad differential. Entities to consider in this differential include carcinomas, lymphomas, neuroblastoma, synovial sarcoma, alveolar rhabdomyosarcoma, Ewing sarcoma, Wilms tumor, rhabdoid tumors, melanoma, desmoplastic small round cell tumor (DSRCT), among others. 

### 6.1. Desmoplastic Small Round Cell Tumor (DSRCT)

Desmoplastic small round cell tumor (DSRCT) is a malignant mesenchymal neoplasm composed of small round tumor cells associated with stromal desmoplasia and a *EWSR1-WT1* gene fusion [1]. Most cases arise in the abdominal cavity with nodules studding the peritoneal surface. Other sites of involvement include the retroperitoneum, pelvis, omentum, and mesentery. DSRCT has a male predominance and primarily affects children and young adults with peak incidence in the third decade of life [1].

Microscopic sections of tumor show a primitive/undifferentiated appearance of monotonous hyperchromatic small round nuclei with minimal cytoplasm, nuclear molding, crush artifact, and indistinct cell borders arranged in well-defined nests separated by a desmoplastic stroma. More rarely, spindled or glandular differentiation can be seen. Central necrosis and frequent mitotic figures are also commonly seen. DSRCT can show a distinctive and complex pattern of multi-phenotypic differentiation, resulting in the expression of epithelial, muscular, and/or neural markers. Most DSRCTs are immunoreactive for various cytokeratins, EMA and desmin (cytoplasmic dot-like pattern). However, other skeletal muscle markers, including myogenin and MYOD1, are negative. Nuclear WT1 staining using an antibody directed to the C-terminus is also usually appreciated. However, this antibody is not available in many pathology laboratories, which normally only carry the WT1 antibody directed to the N-terminus. Molecular studies have consistently characterized a chromosomal translocation t(11;22) between the *EWSR1* gene on 22q12.2 and the Wilms tumor gene, *WT1*, on 11p13 [1]. This fusion can be confirmed by dual break-apart FISH probes for each gene locus and other molecular testing modalities, including next generation sequencing [55]. Despite multimodality therapy, the 5-year overall survival rate is about 10–15% [56].

### 6.2. Ewing Sarcoma

Ewing sarcoma is characterized by a unique gene fusion involving one member of the FET family of genes (usually *EWSR1*) and one member of the ETS family of transcription factors [1]. After osteosarcoma, it is the second most common malignant bone tumor in children and young adults. It is slightly more common in males, with a peak incidence occurring during the second decade of life. Extra-skeletal Ewing’s occurs less frequently in around 12% of cases with most patients being >30 years old at the time of diagnosis. Microscopically, the tumor is composed of small round nuclei with finely stippled chromatin, scant cytoplasm, and indistinct cytoplasmic membranes (Figure 3b). Immunohistochemistry is frequently positive for CD99 (membranous pattern of staining); however, NKX2-2 is a more specific marker [57]. Keratin expression can also be seen in approximately 25% of cases, along with neuroendocrine antigens and/or S-100 protein [58]. Molecular confirmation is often required for the diagnosis of Ewing sarcoma. The most common translocation identified is t(11;22)(q24;q12), which results in the *EWSR1-FLI1* fusion that is seen in about 85% of cases. The second most common is t(21;22)(q22;q12), which results in *EWSR1-ERG* seen in about 10% of cases [1]. Current treatment guidelines for Ewing sarcoma include neoadjuvant chemotherapy and radiation, followed by complete surgical resection and additional adjuvant chemotherapy. The presence of metastases is the most important prognostic factor. Complete pathological response, represented by complete tumor cell necrosis, following neoadjuvant chemotherapy is a favorable prognostic factor [59]. Extra-skeletal Ewing sarcoma occurs in about 12% of patients and has a wide anatomic distribution [1]. Few case reports have documented retroperitoneal Ewing’s sarcoma and, therefore, survival statistics are lacking.

## 7. Solitary Fibrous Tumor (SFT)

SFT is a fibroblastic tumor characterized by a *NAB2-STAT6* gene rearrangement [1]. It occurs equally between males and females with a peak incidence between 40 and 70 years of age. SFT can occur at any anatomic site. Specifically, 30–40% arise in the extremities, 30–40% in the abdomen, pelvis, or retroperitoneum, 10–15% in the head and neck, and 10–15% in the trunk [1]. The tumors are frequently well-circumscribed masses. The cut surface is nodular and occasionally shows hemorrhage, myxoid change, or cystic degeneration. Microscopically, SFT consists of a haphazard arrangement of bland spindle cells with indistinct pale eosinophilic cytoplasm embedded within a variably collagenous stroma, admixed with prominent branching thin-walled dilated vessels (Figure 3h). This architectural vascular pattern is referred to as “stag-horned” or “hemangiopericytoma-like”. The majority of SFTs have low mitotic counts and minimal necrosis or nuclear pleomorphism. There is a wide histological spectrum, ranging from hypocellular lesions, with abundant stromal keloidal-type collagen, to hypercellular tumors with little to no intervening stroma. Other less frequent subtypes include fat-forming (lipomatous) and giant cell-rich SFT. SFTs are typically strongly and diffusely positive for CD34 and nuclear STAT6 (Figure 3i); however, expression may be lost in dedifferentiated tumors. 

The characteristic molecular finding is a paracentric inversion involving chromosome 12q, resulting in a *NAB2-STAT6* fusion [1]. Doege-Potter syndrome is a rare paraneoplastic syndrome that can be observed in patients with SFT in which the tumor produces IGF2 and induces severe hypoglycemia [1]. Although most SFTs have a low metastatic potential, distant or local recurrence can occur in 10–30% of tumors. Rare recurrences are seen after 15 years. A risk stratification model. based on age, size, mitotic index, and necrosis, can help identify patients at high risk for poor outcomes. Specifically, tumors larger than 15 cm, those that occur in patients ≥ 55 years, and those with mitoses ≥4/10 high-power fields, require close follow-up and have a high risk of both metastasis and death [60,61]. The differential diagnosis includes entities such as schwannoma, GIST, MPNST, synovial sarcoma and DDLPS. Rarely, DDLPS may resemble, and be misclassified, as an SFT, posing a potential diagnostic pitfall, particularly in limited tissue biopsies. Specifically, immunohistochemistry on a DDLPS can occasionally detect nuclear STAT6 protein expression. This is because the *STAT6* gene is located near the *MDM2* and *CDK4* genes and is amplified in a subset of DDLPS [62,63].

## 8. Gastrointestinal Stromal Tumor (GIST)

GIST is a mesenchymal neoplasm characterized by differentiation towards the interstitial cells of Cajal [1]. It can occur anywhere along the GI tract and is preferentially seen in the stomach and small bowel. It accounts for 2.2% of all malignant gastric tumors and is slightly more common in men with a peak incidence in the sixth decade of life. Extra-gastrointestinal GIST occurs predominantly in the mesentery, omentum, or retroperitoneum, which likely represents metastasis from an unrecognized primary lesion or a detached mass from another location in the gastrointestinal tract. Most cases are sporadic; however, 5–10% can be seen in syndromes that are frequently succinate dehydrogenase (SDH) deficient. These SDH-deficient GISTs typically occur in younger patients, particularly pediatric patients [1]. Such examples include non-hereditary Carney triad and autosomal dominant Carney-strakis syndrome [64,65]. Tumors are usually well circumscribed and of variable size. The microscopic features frequently show a spindle cell lesion with minimal nuclear pleomorphism; however, an epithelioid morphology can be seen in 20–25% of cases. Immunohistochemistry is usually positive for KIT (CD117), ANO1/DOG1, and CD34. The most common molecular alteration seen in 85% of cases is a gain of function mutation in either the *KIT* or *PDGFRA* oncogenes located on chromosome 4 [1]. Both genes encode type III receptor tyrosine kinases. About 75% of GISTs have activating mutations of *KIT*, most often in exon 11 (66%) or exon 9 (6%); mutations in exons 13 and 17 are uncommon (~1% each). Alternatively, 10% of GISTs have *PDGFRA* activating mutations (most often in gastric GIST), usually in exon 18 (8%). 

The best prognostic factors include mitotic activity, tumor size and anatomical site. Mutation status represents a prognostic and predictive factor. Tumors with *KIT*-mutations tend to behave more aggressively than tumors with *PDGFRA* mutations [1]. Mutation status also predicts response to Imatinib (Gleevec) with *KIT* exon-11 mutants exhibiting the highest response rate and those with *KIT* exon 9 mutations benefiting from higher doses of Imantinib (800 mg vs. 400 mg) [66,67]. Other differential diagnoses to consider include schwannoma, inflammatory fibroid polyp, SFT, poorly differentiated carcinoma, leiomyoma and LMS. 

## 9. Inflammatory Myofibroblastic Tumor (IMT)

IMT is a neoplasm composed of myofibroblastic spindle cells and of borderline malignancy, classically featuring a mixed inflammatory cell population composed of plasma cells, lymphocytes, and/or eosinophils [1]. It usually affects children and young adults and has a slight female predominance. IMT most often arises in the abdomen, including the mesentery, omentum, retroperitoneum, and pelvis; however. other sites can also be involved. Microscopically, the lesion is composed of myofibroblastic spindle cells without overt cytologic atypia in a background of myxoid to collagenous stroma interspersed by a lymphoplasmacytic infiltrate (Figure 3e). Approximately 60% of cases harbor an *ALK* rearrangement that can be detected by immunohistochemistry [1]. Additional stains are variably positive for SMA, MSA, calponin, and desmin and keratin expression may also be seen in 40–70% of cases. *ALK*-negative IMT can harbor *ROS1* gene rearrangements and *ETV6-NTRK3* gene fusions [1]. Prognosis is generally good, however up to 25% of extra-pulmonary cases may recur depending on anatomical site and resectability, and there are rare instances of distant metastasis. *ALK*-negative IMTs may have a higher rate of metastasis, but ALK immunoreactivity does not appear to correlate with local recurrence [1]. Epithelioid inflammatory myofibroblastic sarcoma is a distinct variant that is typically intra-abdominal and is highly aggressive with worse outcomes [1]. The differential diagnosis of a conventional IMT includes desmoid fibromatosis, GIST, WDLPS, DDLPS, and LMS.

## 10. Angiomyolipoma (AML)

Perivascular epithelioid cell tumors (PEComas) are mesenchymal neoplasms that are usually intimately associated with the walls of blood vessel and typically co-express smooth muscle and melanocytic markers [68,69]. Angiomyolipoma (AML) is a specific type of PEComa that arises mainly in the kidney, and, less frequently, from the retroperitoneal soft tissue [70]. Eighty to ninety percent of AMLs are sporadic and mostly occur in women and in older people. Some cases can be identified in patients with tuberous sclerosis with a peak incidence between the third to fourth decade of life and no sex predominance [70]. AMLs are typically solid, well demarcated, and unencapsulated masses that arise within, or adjacent to, the kidney. The cut surface is yellow to white with a whorled appearance. Microscopically, the tumor is composed of a variable triphasic mixture of adipose tissue, spindled and epithelioid smooth muscle cells, and thick-walled blood vessels (Figure 3j). Cases can be classified as “lipoma-like” or “leiomyoma-like”, according to the relative proportions of adipose or smooth muscle components. Immunohistochemistry shows expression of melanocytic markers HMB45, Melan-A, MITF (Figure 3k) and smooth muscle markers SMA and calponin. In addition, stains for CD68, S-100, ER, PR and desmin may also be positive; however, epithelial markers should always be negative [70]. The primary genetic driver for AML is a biallelic inactivation of *TSC2* (encoding hamartin) or *TSC1* (encoding tuberin) which has been found in 94% of cases. Germline mutations in either of these tumor suppressor genes give rise to tuberous sclerosis. Classic AMLs are benign and are rarely associated with complications. The differential diagnosis includes entities such as lipoma, LPS, leiomyoma, and LMS.

## 11. Myelolipoma

Myelolipoma is a benign tumor of the adrenal gland, composed of hematopoietic precursors and mature fat [1]. It typically occurs between the fifth to seventh decade of life with no difference between sexes. Myelolipoma is usually an incidental finding on imaging or at autopsy and is not associated with hematologic disorders. Microscopically, the tumor shows islands of trilineage hematopoiesis, often with markedly increased megakaryocytes interspersed with mature adipocytes (Figure 3l). Larger tumors may show hemorrhage, necrosis, calcification and cysts. No malignant progression has ever been reported. Extra-adrenal myelolipomas are extremely rare. However, they can occasionally occur within the retroperitoneum, particularly in in the presacral region. In such cases careful histologic assessment and *MDM2* testing may be required to distinguish a myelolipoma from a WDLPS [71,72].

## 12. Chordoma

Chordoma is a malignant tumor that recapitulates notochordal differentiation, and usually arises within the axial skeleton. Most cases are conventional (95%), with the remainder being either poorly differentiated or dedifferentiated [1]. The anatomic distribution is relatively equal between the skull base, mobile spine, and sacrum/coccyx. Those arising in the sacrum/coccyx or the lower spine may bulge into the retroperitoneal space. All ages are affected, with a peak incidence between the fifth to seventh decade of life and a slight male predominance [1]. Rare cases are associated with a germline tandem duplication of the *TBXT* gene [73]. Chordoma can also arise in the setting of tuberous sclerosis via loss of function of the *TSC1* or *TSC2* tumor suppressor genes [74]. Microscopically, conventional chordoma is composed of large epithelioid cells with pale eosinophilic cytoplasm arranged in chords or nests within a background of myxoid stroma. Physaliphorous cells are pathognomonic for chordoma and can be identified by their bubbly cytoplasm. Variable degrees of nuclear atypia and mitotic figures can also be seen. Immunohistochemistry is usually diffusely positive for cytokeratin, EMA, and nuclear brachyury with variable S-100 positivity. Molecular studies have identified T gene (brachyury) duplication (6q27) in approximately 27% of sporadic chordomas; however, nearly all notochordal tumors overexpress brachyury [75,76]. Expression of brachyury can, therefore, help distinguish chordoma from other entities, such as chondrosarcoma, chordoid meningioma, metastatic carcinoma and myoepithelial tumors. Overall, the median survival is 7 years, and approximately 40% of chordomas that arise at sites other than the base of the skull metastasize [1].

## 13. Angiosarcoma

Angiosarcomas are rare, accounting for approximately 2% to 4% of all soft tissue sarcomas [77,78]. More than 50% of angiosarcomas arise in the skin, while the remainder frequently occur in the breast, visceral organs, or deep soft tissues, such as the deep muscles of the lower extremity [79]. Primary angiosarcoma of the retroperitoneum is exceedingly rare and the literature of these tumors is limited [79,80,81,82,83,84].

Most often, the etiology of an angiosarcoma is unknown. Some cases arise following radiation exposure or chronic lymphedema [1]. There are rare reported associations of angiosarcomas arising in the setting of implanted foreign materials, in association with arteriovenous fistulas or vascular lesions, including vascular malformations [81,85]. Rarely, angiosarcomas may develop as a heterologous component in other tumors, including nerve sheath tumors (see section on MPNST) and germ cell tumors [86,87]. 

Microscopically, angiosarcomas are malignant vascular neoplasms composed of atypical endothelial cells with infiltrative, poorly defined margins. They have a broad morphological appearance, ranging from lesions that are cytologically bland with well-formed, anastomosing vessels, to solid sheets of highly pleomorphic tumor cells, that may be epithelioid or spindled, without definite vasoformation (Figure 3n). Tumors with high-grade morphology typically show increased mitotic activity, atypical mitotic figures, and coagulative necrosis. By immunohistochemistry, angiosarcomas demonstrate a vascular phenotype with staining for CD31 (membranous), ERG (nuclear), CD34 and Factor VIII. Epithelioid markers, including cytokeratins and EMA, may be positive in angiosarcomas with epithelioid morphology (often designated as epithelioid angiosarcoma). In angiosarcomas that develop in the setting of chronic lymphedema or radiation, there is usually strong positivity for MYC. The latter correlates with *MYC* gene amplifications, which are rarely present in primary angiosarcoma [88,89]. In addition, they may also show alterations in *TP53* and mTOR pathways [90]. Overall, the molecular profile of angiosarcomas typically demonstrate complex karyotypes [91].

## 14. Conclusions

Retroperitoneal sarcomas are relatively rare tumors. Complete surgical resection remains the standard of care. The most frequent types include WDLPS, DDLPS, and LMS. Accurate pathologic diagnosis requires careful histologic examination with the integration of immunohistochemistry, cytogenetics, and molecular studies. Limited tissue biopsies can pose a diagnostic challenge to the pathologist. Specifically, the essential histologic features of a tumor may not be captured, due to sampling bias leading to an incorrect diagnosis or grade, until the excised specimen has been fully sampled. Additionally, WDLPS can mimic benign entities, such as AML, myelolipoma, or leiomyoma; therefore, careful interpretation is required for small biopsies. Overall, a multidisciplinary approach is crucial to arrive at a correct diagnosis.
